# Heavy metals in drinking water and periodontitis: evidence from the national oral health survey from China

**DOI:** 10.1186/s12889-023-16391-3

**Published:** 2023-09-04

**Authors:** Shuduo Zhou, Wenjing Li, Jun Wan, Yixuan Fu, Hongye Lu, Na Li, Xu Zhang, Yan Si, Xing Wang, Xiping Feng, Baojun Tai, Deyu Hu, Huancai Lin, Bo Wang, Chunxiao Wang, Shuguo Zheng, Xuenan Liu, Wensheng Rong, Weijian Wang, Xuliang Deng, Zhenyu Zhang

**Affiliations:** 1 Department of Global Health, School of Public Health, Peking University, Beijing, China; 2 Institute for Global Health and Development, Peking University, Beijing, China; 3 Beijing Laboratory of Biomedical Materials, Department of Geriatric Dentistry, Peking University School and Hospital of Stomatology, Beijing, 100081 China; 4 Institute of Medical Technology, Peking University Health Science Center, Beijing, 100191 P. R. China; 5 Department of Orthodontics, School and Hospital of Stomatology, Peking University, Beijing, China; 6 Department of prosthodontics, Hebei Key Laboratory of Stomatology, Hebei Clinical Research Center for Oral Diseases, School and Hospital of Stomatology, Hebei Medical University, Shijiazhuang, China; 7 Stomatology Hospital, School of Stomatology, Dental Biomaterials and Devices for Zhejiang Provincial Engineering Research Center, Zhejiang Provincial Clinical Research Center for Oral Diseases, Key Laboratory of Oral Biomedical Research of Zhejiang Province, Zhejiang University School of Medicine, Cancer Center of Zhejiang University, Hangzhou, China; 8 Peking Union Medical College Hospital, Chinese Academy of Medical Science & Peking Union Medical College, Beijing, China; 9 Department of Preventive Dentistry, National Engineering Laboratory for Digital and Material Technology of Stomatology, Beijing Key Laboratory of Digital Stomatology, Peking University School and Hospital of Stomatology, Beijing, China; 10 Chinese Stomatological Association, Beijing, P.R. China; 11 Shanghai Ninth People’s Hospital, Shanghai Jiao Tong University School of Medicine, Shanghai, P.R. China; 12 School & Hospital of Stomatology, Wuhan University, Wuhan, P.R. China; 13 West China School of Stomatology, Sichuan University, Chengdu, P.R. China; 14 Guanghua School of Stomatology, Hospital of Stomatology, Sun Yetsen University, Guangzhou, P.R. China; 15 Chinese Center for Disease Control and Prevention, Beijing, P.R. China

**Keywords:** Drinking water, Essential trace heavy metals, Periodontitis, Survey

## Abstract

**Background:**

Periodontitis has become an increasingly important public health issue, coupled with a high economic burden for prevention and treatment. Exposure to essential trace heavy metals has been associated with various diseases; however, the relationships between essential trace heavy metals and periodontitis remain inconclusive.

**Objectives:**

To investigate the association between essential trace heavy metals in tap water and periodontitis in a nationally representative sample in China.

**Methods:**

We conducted a nationwide study including 1348 participants from the Fourth National Oral Health Survey in the 2015–2016 period. The trace heavy metals concentration was measured in the local pipeline terminal tap water. Periodontitis was diagnosed according to the classification scheme proposed at the 2018 world workshop on the classification of periodontal and peri-implant diseases and conditions. We used weighted multivariable logistic regression to estimate the association between essential trace heavy metals and the risk of periodontitis. We additionally used spline analysis to explore the possible nonlinear dose-response associations.

**Results:**

Periodontitis patients were exposed to higher concentrations of essential trace heavy metals. In adjusted models, for 1 SD increase in the concentration of iron, manganese, and copper in tap water, the risk of periodontitis increased by 30% (OR: 1.30, 95%CI: 1.12–1.50), 20% (OR: 1.20, 95%CI: 1.03–1.41), and 20% (OR: 1.20, 95%CI: 1.04–1.39), respectively. Stratified analyses demonstrated that the associations between essential trace heavy metals and periodontitis were higher in females, elders, and rural residents. Spline analysis revealed nonlinear exposure-response relationships between periodontitis and exposure to iron, manganese, and copper in tap water.

**Conclusions:**

Exposures to essential trace heavy metals in drinking water were associated with greater odds of periodontitis. Given the growing burden of periodontitis, our study sheds light on tailored public health policies for improving drinking water standards to alleviate periodontitis impairment.

## Introduction

Periodontitis is a chronic and long-lasting inflammatory condition stemming from the synthetic effects of genes, social and behavioral factors, and the environment [1, 2]. Periodontitis has been an important public health issue with high prevalence and association with systemic diseases, such as rheumatoid, coronary artery disease, and diabetes [3, 4]. It was estimated that the prevalence of periodontitis ranges from 20–50% [5], and countries with lower economic levels had a higher rate [6, 7]. According to World Health Organization statistics, severe periodontal diseases affect more than one billion people worldwide [8], accounting for more than 3.5 million years lived with disability and causing more than $54 billion in global costs each year [9, 10]. In China, the prevalence and burden of periodontitis are increasing with the rapid progress of aging, and nearly 90% of Chinese adults suffer from varying degrees of periodontal diseases [11, 12].

A growing number of epidemiologic studies have suggested that tobacco use, poor oral hygiene, nutrition, poor socioeconomic conditions, air pollution exposure, and aging are important collective risk factors for periodontitis [13–17]. Some observational studies have shown associations between heavy metals and periodontitis, such as cadmium and lead, which may disrupt bone remodeling and induce oxidative stress [18, 19]. Trace heavy metals such as iron, zinc, copper, and manganese are essential elements related to human health. However, exposure to high levels of essential heavy metals has shown relationships with cancers and other acute diseases [20, 21]. Clinical case-control studies indicated that serum zinc and copper levels were higher in teeth with periodontitis than healthy teeth [22–24].

Research about exposures to trace heavy metals and periodontitis is scarce. Thus, we hypothesize that exposure to a higher level of trace heavy metals may increase the risk of periodontitis. To fill the knowledge gap, we estimate the association between exposure to trace heavy metals (iron, manganese, copper, and zinc) in drinking water and the risk of periodontitis in national representativeness. We also identified the susceptible population and examined the dose-response relationship.

## Materials and methods

### Study population

The National Oral Health Survey of China (CNOHS) is a nationally representative oral health survey and oral clinical examination assessing the oral health status of Chinese children and adults. The fourth CNOHS used a multistage cluster sampling method, and a more detailed description of the study design and sampling procedure was published elsewhere [11, 25]. A total of 124 districts (91 cities) were selected from 31 provinces using the probability-proportional-to-size (PPS) method, considering the variation in population size. From these districts, participants between 35 and 74 years old were chosen from 372 communities (three communities from each urban and rural district) using quota sampling. In this study, due to the availability of water quality data, and the periodontal examinations only conducted in adults, we adopted the study participants to those 35–75 years of age from selected geographic and cultural representative cities. Therefore, our study population includes 1,348 participants, which represent over forty million adults from twelve counties/districts in six provinces. The Chinese Stomatological Association Ethics Committee approved this study (Approval no. 2014-003). Written informed consent from participants was obtained before they completed the questionnaires.

### Exposure assessment

Two chlorinated water samples (dry [May] and wet [September] seasons) prior to the oral examination date were collected from the pipeline terminal close to the participants’ residential addresses. The local Center for Disease Control and Prevention analyzed the terminal tap water samples for physicochemical indices. The trace heavy metals, including iron, manganese, copper, and zinc were measured by atomic absorption spectrophotometry or atomic absorption method. During the analysis, replicates were introduced to ensure the accuracy of the analysis. The relative errors of the replicates were within ± 5%, indicating acceptable analytical accuracy. To aid in the comparison of exposure test results, we calculated standardized Z scores scaled to the regional measurements by subtracting the regional measurement mean from each measurement site’s test values and dividing by the regional measurement standard deviation (SD) for those tests with approximately Gaussian distributions (manganese, iron, copper, and zinc).

### Periodontitis measurement

Well-trained dentists performed periodontal examinations using a community periodontal index probe (CPI). A calibration training program was conducted before the survey to ensure the reliability of the results. In the survey, 5% of the random participants were selected to test the inter-examiner reproducibility. The kappa values for the three age groups ranged from 0.76 to 0.8. Four periodontal parameters, including bleeding on probing (BOP), presence of calculus, periodontal probing depth (PPD), and clinical attachment loss (CAL) were assessed. The diagnosis of periodontitis is according to the classification scheme proposed at the 2018 World Workshop on the Classification of Periodontal and Peri-Implant Diseases and Conditions [1]. Participants with < 10% BOP-positive teeth and PPD ≤ 3 mm were classified as periodontal healthy. Periodontitis was classified to four stages depending on the four periodontal parameters. A CAL of 1–2 mm was classified as a stage I periodontal patient, a CAL of 3–4 mm was classified as a stage II periodontal patient, CAL over 5 mm was classified as a stage III periodontal patient, CAL over and equal 5 mm and with over and equal 5 missing teeth was classified as stage IV periodontal patient. Stage II periodontal patients were reclassified as stage III when the maximum PPD was over and equaled 6 mm [11]. In this study, periodontitis patients were diagnosed with stage I to IV periodontitis.

### Covariates

We selected potential confounders based on a literature review of previous studies. All the variables were collected using a standard structured questionnaire through face-to-face interviews by trained investigators. Potential risk factors about sociodemographic status, health behavior, and health conditions were controlled in our study. The sociodemographic status included age (35–49, 50–64, or 65–74), sex (male versus female), ethnicity (Han versus others), educational attainment (illiteracy, middle school and below, high school, or college), and residence status (living in rural versus urban areas). Health behavior covariates were measured by smoking status (never, former, or current), alcohol drinker(never, seldom, frequent, or former), frequency of having dessert or beverage (seldom, sometimes, or frequent), and whether the participants did teeth cleaning by dentists in the past 12 months (yes versus no). Covariates for health status included a history of stroke, diabetes, hypertension, heart disease, and chronic obstructive pulmonary disease (COPD) (yes versus no).

### Statistical analysis

Descriptive analyses were conducted for all variables. We used weighted multivariable logistic regression models to estimate essential trace heavy metals exposure in drinking water and the risk of periodontitis with national representativeness. This study specified two models to adjust for a set of variables considered as risk factors. In the basic model (model 1), we adjusted for age, sex, ethnicity, educational attainment, and residence status. In the fully adjusted model (model 2), we further adjusted for current smoking status, alcohol consumption status, frequency of desserts or beverages, underwent teeth cleaning performed by dentists within the past 12 months, and medical history of stroke, diabetes, hypertension, heart diseases, and COPD. We conducted stratification analyses to explore whether the associations could be modified by sex, age, living region, smoking status, alcohol consumption, dessert consumption, and beverage consumption by including interaction terms between exposures and potential modifiers. For each potential effect modifier, we evaluated effect modification by likelihood ratio tests comparing models that included an interaction (product) term between essential trace heavy metals exposure and the effect modifier versus models without the interaction term. Stratum-specific ORs were obtained from the same interaction model by using the appropriate coefficients and variance-covariance matrix.

In the spline analysis, we explore the dose-response associations between essential trace heavy metals and the risk of periodontitis. All associations are presented as ORs with corresponding 95% CIs. A 2-sided p-value < 0.05 was considered statistically significant. Stata version 16.0 for Mac (Stata Corp, College Station, TX, USA) and R Studio Version 1.2.5042 (The R Project for Statistical Computing, Vienna, Austria) were used for the statistical analyses.

## Results

Compared with healthy participants, participants with periodontitis tended to be male (55.6% versus 43.1%, *p* < 0.001), middle-aged and older (73.1% versus 59.6%, *p* < 0.001), smokers, alcohol drinkers, and have a lower level of educational attainment (Table 1). The concentration of iron, manganese, and copper in drinking water for participants with periodontitis was higher than for healthy participants.

**Table 1:** Characteristics of study participants

	Healthy	Periodontitis	*p value* ^*a*^
	591	757	
Age, y (%)			< 0.001
35–49	239 (40.4)	204 (26.9)	
50–64	173 (29.3)	280 (37.0)	
65–74	179 (30.3)	273 (36.1)	
Female sex (%)	336 (56.9)	344 (45.4)	< 0.001
Han ethnicity (%)	589 (0.3)	753 (0.5)	0.91
Education (%)			< 0.001
Illiteracy	67 (11.3)	111 (14.7)	
Middle School	301 (50.9)	411 (54.3)	
High School	104 (17.6)	149 (19.7)	
College	119 (20.1)	86 (11.4)	
Live in Rural Area (%)	159 (26.9)	164 (21.7)	0.03
Iron^*^	0.065 (0.04)	0.074 (0.04)	< 0.001
Manganese^*^	0.031 (0.01)	0.033 (0.01)	0.07
Copper^*^	0.045 (0.04)	0.051 (0.04)	0.001
Zinc^*^	0.043 (0.02)	0.043 (0.02)	0.91
Smoke (%)			0.001
Never smoked	408 (69.0)	452 (59.7)	
Current smokes	121 (20.5)	215 (28.4)	
Former smoked	62 (10.5)	90 (11.9)	
Alcohol (%)			0.001
Never	342 (57.9)	377 (49.9)	
Seldom	133 (22.5)	161 (21.3)	
Frequent	93 (15.7)	179 (23.7)	
Former	23 (3.9)	39 (5.2)	
Frequent to have Dessert (%)			0.03
Seldom	400 (67.7)	530 (70.0)	
Sometimes	136 (23.0)	135 (17.8)	
Frequent	55 (9.3)	92 (12.2)	
Frequent to have Beverage (%)			0.76
Seldom	522 (88.3)	668 (88.2)	
Sometimes	51 (8.6)	61 (8.1)	
Frequent	18 (3.0)	28 (3.7)	
Clean Teeth (%)	562 (95.1)	726 (95.9)	0.56
Comorbidity (%)			
Stroke	8 (1.4)	7 (0.9)	0.63
Diabetes	55 (9.3)	77 (10.2)	0.66
Hypertension	157 (26.6)	248 (32.8)	0.02
Heart Diseases	46 (7.8)	67 (8.9)	0.55
COPD	8 (1.4)	14 (1.8)	0.62

Abbreviations: COPD, chronic obstructive pulmonary disease. Continuous variables are described as the mean ± standard deviation (SD), and categorical variables are expressed as counts and percentages

^*^Mean ± standard deviation (SD),

^*a*^ Differences in the distribution between healthy and periodontitis participants were tested using the Mann‒Whitney U test for continuous variables and the chi-square test for categorical variables

In the fully adjusted model, exposure to a higher concentration of iron, manganese, and copper in drinking water was significantly associated with a higher risk of periodontitis (Table 2). For 1 SD increase in the concentration of iron, manganese, and copper in drinking water, the risk of periodontitis increased by 30% (OR: 1.30, 95%CI: 1.12–1.50), 20% (OR: 1.20, 95%CI: 1.03–1.41), and 20% (OR: 1.20, 95%CI: 1.04–1.39), respectively. There was no significant association between exposure to zinc in drinking water and periodontitis, with an OR of 0.98 (95%CI: 0.86–1.12).

**Table 2:** Association between essential trace heavy metals and periodontitis in the national fourth oral health survey

	Periodontitis
Iron	
Model 1	1.27 (1.10, 1.46)
Model 2	1.30 (1.12, 1.50)
Manganese	
Model 1	1.17 (1.00, 1.36)
Model 2	1.20 (1.03, 1.41)
Copper	
Model 1	1.17 (1.01, 1.35)
Model 2	1.20 (1.04, 1.39)
Zincs	
Model 1	0.97 (0.86, 1.11)
Model 2	0.98 (0.86, 1.12)

Model 1 adjusted for age, sex, ethnicity, education, and urban or rural areas. Model 2 further adjusted for current smoking status, current alcohol consumption status, frequency of sweet desserts, frequency of beverages, cleaning the tooth in the past 12 months, medical history of stroke, diabetes, hypertension, heart diseases, and COPD. Data are presented as percentages (95% CI).

Stratified analyses demonstrated that the associations between essential trace heavy metals and periodontitis were higher in females, elders, and rural residents (Table 3). With 1 SD increase in exposure to iron in tap water, the risk for periodontitis increased by 39% (OR: 1.39, 95%: 1.14–1.68) in the female participants and 22% (OR: 1.22, 95%: 0.99–1.50) in male participants, 34% (OR: 1.34, 95%: 1.06–1.68) for the older participants and 23% (OR: 1.23, 95%: 0.98–1.54) for the younger, and 37% (OR: 1.37, 95%: 1.11–1.70) for the residents in rural areas while 28% (OR: 1.28, 95%: 1.07–1.53) for the participants in urban areas. Similar results were found in the stratified analyses of the associations between manganese, copper, and periodontitis.

**Table 3:** Associations between essential trace heavy metal elements and periodontitis by different characteristics of participants in the national fourth oral health survey

	Iron	Manganese	Copper	Zinc
**Sex**				
Male	1.22 (0.99, 1.50)	1.16 (0.94, 1.44)	1.16 (0.94, 1.42)	0.98 (0.81, 1.19)
Female	1.39 (1.14, 1.68)	1.25 (1.03, 1.53)	1.25 (1.03, 1.52)	0.99 (0.83, 1.18)
**Age, yrs**				
35–49	1.34 (1.07, 1.66)	1.25 (0.99, 1.58)	1.21 (0.97, 1.51)	1.00 (0.82, 1.22)
50–64	1.23 (0.98, 1.54)	1.10 (0.88, 1.36)	1.17 (0.94, 1.46)	0.99 (0.80, 1.22)
65–74	1.34 (1.06, 1.68)	1.31 (1.05, 1.65)	1.25 (1.00, 1.57)	0.94 (0.76, 1.16)
**Living region**				
Urban	1.28 (1.07, 1.53)	1.18 (1.01, 1.39)	1.17 (1.00, 1.37)	1.04 (0.90, 1.19)
Rural	1.37 (1.11, 1.70)	2.02 (1.23, 3.32)	1.57 (1.15, 2.15)	0.60 (0.43, 0.86)
**Educational level**				
Illiteracy	1.26 (0.93, 1.71)	1.32 (0.92, 1.89)	1.26 (0.88, 1.82)	1.00 (0.74, 1.34)
Middle School	1.23 (1.02, 1.47)	1.10 (0.91, 1.33)	1.12 (0.94, 1.34)	1.07 (0.89, 1.29)
High School	0.99 (0.71, 1.38)	0.99 (0.71, 1.38)	0.89 (0.65, 1.22)	1.33 (0.97, 1.82)
College	2.93 (1.62, 5.32)	1.89 (1.25, 2.84)	2.23 (1.40, 3.55)	0.54 (0.38, 0.76)
**Frequency of dessert consumption**				
Seldom	1.29 (1.10, 1.51)	1.22 (1.02, 1.46)	1.21 (1.02, 1.42)	0.95 (0.82, 1.11)
Sometime	1.54 (1.08,2.21)	1.32 (0.97, 1.82)	1.36 (0.98, 1.90)	1.02 (0.76, 1.36)
Frequent	0.96 (0.56, 1.62)	0.88 (0.57, 1.36)	0.86 (0.54, 1.37)	1.25 (0.80, 1.96)
**Frequency of beverage consumption**				
Seldom	1.31 (1.13, 1.52)	1.21 (1.03, 1.43)	1.22 (1.05, 1.42)	0.96 (0.84, 1.10)
Sometime	1.45 (0.77, 2.73)	1.34 (0.80, 2.25)	1.28 (0.68, 2.38)	1.24 (0.78, 2.00)
Frequent	0.91 (0.33, 2.51)	1.00 (0.42, 2.39)	0.87 (0.37, 2.05)	1.15 (0.45, 2.97)
**Smoke**				
Never	1.31 (1.10, 1.56)	1.21 (1.00, 1.45)	1.18 (0.99, 1.41)	1.00 (0.85, 1.18)
Current	1.40 (1.04, 1.88)	1.30 (0.98, 1.73)	1.32 (0.98, 1.79)	1.01 (0.78, 1.30)
Former	1.09 (0.73, 1.63)	1.03 (0.68, 1.55)	1.11 (0.73, 1.68)	0.83 (0.56, 1.25)
**Frequency of alcohol consumption**				
Never	1.36 (1.13, 1.65)	1.27 (1.05, 1.55)	1.27 (1.05, 1.54)	1.09 (0.91, 1.31)
Seldom	1.07 (0.79, 1.46)	1.03 (0.78, 1.36)	1.01 (0.76, 1.36)	0.84 (0.65, 1.08)
Frequent	1.28 (0.94, 1.75)	1.19 (0.87, 1.63)	1.17 (0.84, 1.62)	1.08 (0.81, 1.44)
Former	2.39 (2.39, 5.29)	2.04 (0.94, 4.41)	1.95 (0.86, 4.43)	0.63 (0.32, 1.25)

Abbreviations: COPD, chronic obstructive pulmonary disease

Models were adjusted for age, sex, ethnicity, education, living in urban or rural areas, current smoking status, current alcohol consumption status, frequency of sweet dessert, frequency of beverages, clean teeth in the past 12 months, medical history of stroke, diabetes, hypertension, heart diseases, and COPD

**Fig. 1:**
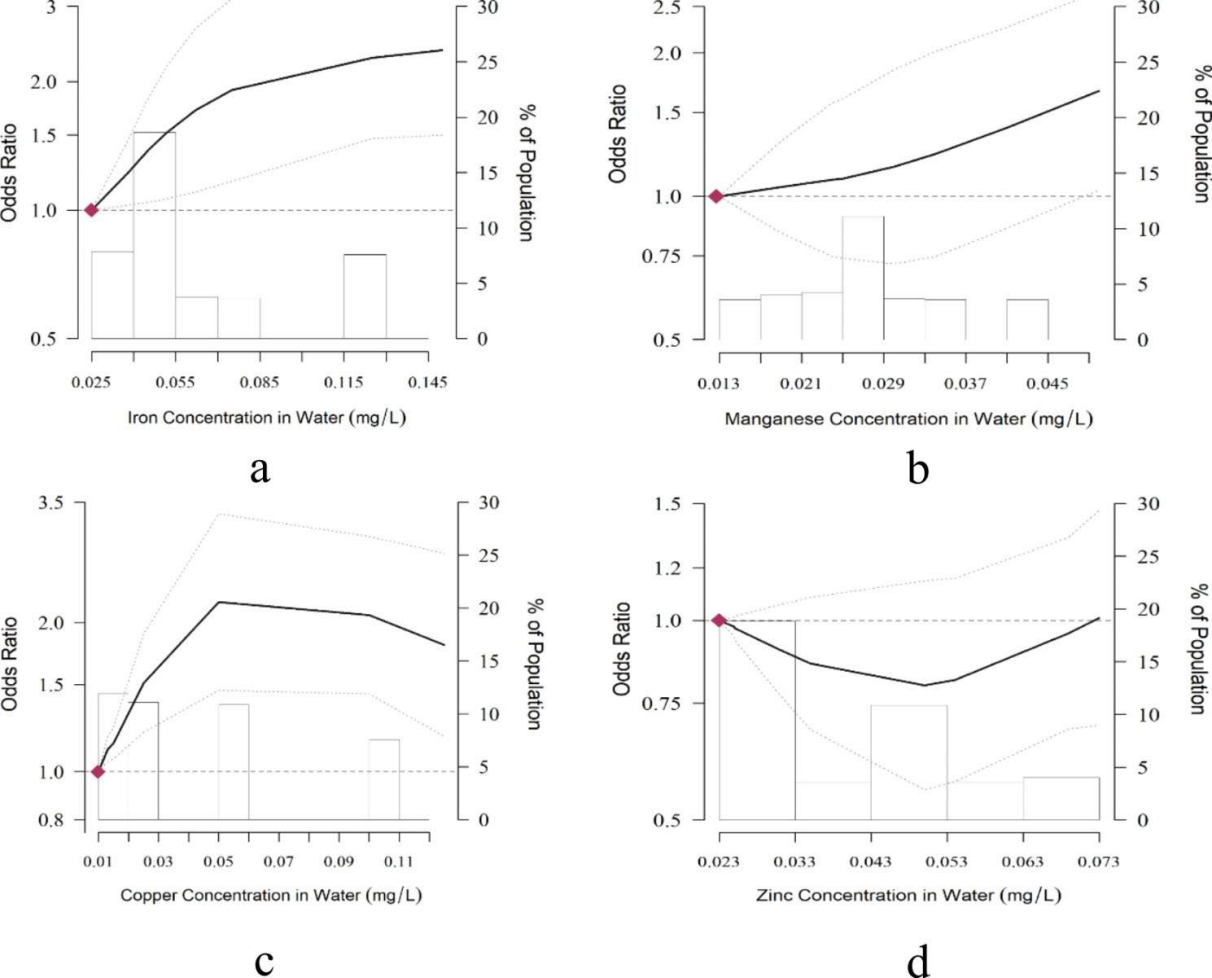
Odds Ratios (ORs) for Risks of Periodontitis by the Level of Exposure to Essential Trace Heavy Metals Concentrations in Water **a**: iron, **b**: manganese, **c**: copper, **d**: zinc The dose‒response curve was calculated using restricted cubic splines with knots at the 25th, 50th, and 95th percentiles of the distribution of the 12-month Iron concentration in Water. The reference exposure level was set at the 5th percentile of the distribution of the 12-month iron concentration (0.0249 mg/L), manganese concentration (0.0127 mg/L), copper concentration (0.0097 mg/L) and zinc concentration (0.0230 mg/L). ORs were adjusted for age, sex, ethnicity, education, living in urban or rural areas, current smoking status, current alcohol consumption status, frequency of sweet dessert consumption, frequency of beverages, tooth cleaning in the past 12 months, medical history of stroke, diabetes, hypertension, heart diseases, and COPD.

There were no significant modification effects on the associations between essential trace heavy metals and periodontitis between different socioeconomic statuses and health behavior groups. Spline analysis revealed nonlinear exposure-response relationships between periodontitis and exposure to iron, manganese, and copper in tap water (Fig. 1). Exposure to iron in tap water significantly increased the risk of periodontitis with larger concentrations associated with higher risks. Exposure to a high level of manganese considerably raised the risk of periodontitis; however, low-dose manganese exposure had no significant influence on increased risk. There was a potential threshold effect between copper exposure in tap water and periodontitis, and when the copper concentration exceeded 0.05 mg/L, the risk value decreased but remained significant.

## Discussion

Leveraging this nationwide study, we estimated the association between trace heavy metals in tap water and periodontitis. We found that exposure to iron, manganese, and copper was associated with higher risks of periodontitis, and the associations were more pronounced in females, elders, and rural residents. Our study highlighted the potential role of reducing exposure to trace heavy metals in drinking water to alleviate the disease burden of periodontitis, especially in countries that are progressing into an aging society.

The underlying mechanisms of these associations are less clear. Trace heavy metals are absorbed through various pathways, such as the gastrointestinal macro, and transported through the blood to various tissues [26]. Previous studies pointed out that dentin could be used as a marker of environmental metal exposure [27]. Trace heavy metal exposure affects the structure and function of the salivary microbiota in the oral cavity, which is an important determinant of dental caries and periodontal disease [28]. In addition, excessive copper accumulation could destroy the periodontal tissue and potentially accelerate the process of tissue destruction via an inflammatory response [29]. Bone is the primary storage organ for manganese in the human body, and excessive accumulation of manganese has adverse effects on the structure and function of bones [30], which may destabilize the alveolar bone and potential explain our findings. In addition, we found no significant association between exposure to zinc and periodontitis. However, the associations were inconsistent among animal and human studies [31, 32]. Prospective cohort studies are needed to test our hypothesis.

Studies have shown inequalities in periodontal health, and people with lower socioeconomic status suffer from a higher burden of periodontal diseases [33, 34]. In the current study, we further suggested a higher association between exposure to essential trace heavy metals such as iron and copper and the development of periodontitis in women, older adults, and people in rural areas. The potential explanations for the findings include in vivo exposure level and dental services utilization. Additionally, the drinking water quality problem in rural areas [35], and dental services utilization inequalities between rural and urban areas [36], may amplify the impact of trace heavy metals on periodontitis. Achieving health equity is the core goal of universal health coverage. Reducing the levels of essential trace heavy metals in drinking water could improve periodontal health, especially in vulnerable groups such as women, older adults, and people in rural areas, narrowing periodontal health inequalities.

Safe drinking water is what people need to survive, and this study suggested that prolonged exposure to trace heavy metals could increase the risk of periodontitis. Furthermore, we found a potential threshold effect between copper exposure in drinking water and the prevalence of periodontitis. In China, the existing drinking water standards were issued ten years ago [37], and the new standards will come into force in April 2023. We propose establishing a dynamic update mechanism for drinking water standards based on the latest research on the relationship between drinking water and health. More prospective studies are needed to produce more robust evidence to drive further improvements in Chinese drinking water standards.

Several limitations should be acknowledged. First, the observational nature of this study limited our ability to infer a causal relationship between periodontitis and exposure to trace heavy metals in drinking water. Second, our study’s exposures to essential trace heavy metals were at the district/county level rather than the individual level. Individual exposure levels may vary depending on drinking habits and the volume. Third, as this was a national survey nature, we could not analyze whether the levels of iron, manganese, and copper in periodontal tissues were elevated or specifically because of water exposure.

Fourth, although we have adjusted for some covariates in models, other potential factors including dietary patterns may distort the results. In terms of strengths, this study is the first to examine the association between essential trace heavy metals exposure and risk of periodontitis with national representativeness in low- and middle-income countries. The clinical diagnosis of periodontitis and professional exposure measurement alleviated the measurement bias. Our results might also provide an essential reference for other middle-income countries, given the large diversity in the selected geographically represented cities. Finally, this study followed strict quality control methods in data collection, collation, and data analysis to ensure the scientific validity of the results.

## Conclusions

This nationally representative ecological study found that exposure to essential trace heavy metals in drinking water was associated with greater odds of periodontitis. Given the growing burden of periodontitis, our study sheds light on tailored public health policies for improving drinking water standards to alleviate periodontitis impairment. Further studies are warranted to explore the multiple mechanisms and causal relationships between exposure to essential trace heavy metal elements and periodontitis.

## Data Availability

The datasets used and/or analysed during the current study available from the corresponding author on reasonable request.
